# Dopamine: from prediction error to psychotherapy

**DOI:** 10.1038/s41398-020-0814-x

**Published:** 2020-05-25

**Authors:** Silvia Papalini, Tom Beckers, Bram Vervliet

**Affiliations:** 1 Laboratory of Biological Psychology (LBP), Faculty of Psychology and Educational Sciences, KU Leuven, Leuven, Belgium; 2 Leuven Brain Institute, KU Leuven, Leuven, Belgium; 3 Centre for the Psychology of Learning and Experimental Psychopathology (CLEP), Faculty of Psychology and Educational Sciences, KU Leuven, Leuven, Belgium

**Keywords:** Physiology, Neuroscience

## Abstract

Dopamine, one of the main neurotransmitters in the mammalian brain, has been implicated in the coding of prediction errors that govern reward learning as well as fear extinction learning. Psychotherapy too can be viewed as a form of error-based learning, because it challenges erroneous beliefs and behavioral patterns in order to induce long-term changes in emotions, cognitions, and behaviors. Exposure therapy, for example, relies in part on fear extinction principles to violate erroneous expectancies of danger and induce novel safety learning that inhibits and therefore reduces fear in the long term. As most forms of psychotherapy, however, exposure therapy suffers from non-response, dropout, and relapse. This narrative review focuses on the role of midbrain and prefrontal dopamine in novel safety learning and investigates possible pathways through which dopamine-based interventions could be used as an adjunct to improve both the response and the long-term effects of the therapy. Convincing evidence exists for an involvement of the midbrain dopamine system in the acquisition of new, safe memories. Additionally, prefrontal dopamine is emerging as a key ingredient for the consolidation of fear extinction. We propose that applying a dopamine prediction error perspective to psychotherapy can inspire both pharmacological and non-pharmacological studies aimed at discovering innovative ways to enhance the acquisition of safety memories. Additionally, we call for further empirical investigations on dopamine-oriented drugs that might be able to maximize consolidation of successful fear extinction and its long-term retention after therapy, and we propose to also include investigations on non-pharmacological interventions with putative prefrontal dopaminergic effects, like working memory training.

## Introduction

It is becoming increasingly clear that prediction errors have a central role in the shaping of our actions and expectations^1,2^. Prediction errors occur when there is a mismatch between the expected state and the actual state of the world: they serve as a signal that current expectations are inaccurate and should be updated. Formalized theories of learning specify how prediction errors govern the updating of expectations in a changing environment, and decades of experimental research have established a major role for dopamine signaling in this error-based learning process and in keeping updated expectations and other mental representations stable over time^3^. Thus, dopamine is emerging as a central neuromodulator in the flexible guidance of adaptive behavior.

Psychopathology, on the other hand, is typically characterized by maladaptive behaviors that are inflexible and resistant to change. Recent theories in this domain propose that dysfunctional expectations are at the heart of maladaptive behaviors and it is argued that effective psychotherapies work by violating and updating such dysfunctional expectations^4–6^. In this review, we propose that the violation of dysfunctional expectations in psychotherapy shares important similarities with dopamine-based prediction errors. This approach lays the ground for a mechanistic understanding of psychotherapy in terms of formal learning theory and cognitive neuroscience. In addition, because dopamine is also involved in keeping updated expectations stable over time, we propose that its role may expand to maintaining treatment gains over the long-term and decreasing the risk of relapse.

We start this review by discussing the role of dysfunctional expectations in psychopathology and the importance of expectancy violation for psychotherapeutic change. We then elaborate on the proposed link between expectancy violations and prediction errors, and we review the evidence for an involvement of mesolimbic dopamine signaling. As a case in point, we emphasize recent work on the involvement of dopamine-based prediction errors in the acquisition of fear extinction as a model of exposure-based psychotherapy for pathological anxiety based on safety learning. We furthermore report evidence that hippocampal and prefrontal dopamine is important for the consolidation of fear extinction memories. Finally, we integrate several lines of research on meso-cortical dopamine signaling, fear extinction, and working memory, and we propose that non-pharmacological interventions such as working memory training should be considered in future empirical research as a way to modulate dopamine levels and contribute to long-term gains of psychotherapy.

## Dysfunctional expectations are at the heart of many mental disorders

Learning to predict when important events will occur is crucial for survival. Accurate expectations about rewards guide appropriate approach behaviors to collect and consume recompenses, while accurate expectations about threat guide appropriate avoidance behaviors to prevent dangerous encounters. Many of these expectations are triggered by cues or actions that reliably preceded important outcomes in the past. Arguably, these past experiences laid down in memory an association between the cue/action and the outcome, so that future occurrences of the cue/action trigger the expectation of the outcome. The challenge for adaptive learning is to arrive at accurate expectation values from only a limited set of contingency experiences. Sometimes, this expectation learning process goes awry. In anxiety patients, erroneous expectations of danger trigger excessive fear levels^7,8^ and motivate disabling avoidance behaviors^9^. For example, following an embarrassing moment in a group conversation, a socially anxious individual may develop an exaggerated expectation that conversations lead to embarrassment, and therefore avoid speaking up during group conversations. Depressed patients, on the other hand, have pessimistic expectations about their self and their future^5^. Experiences of failures lead to the exaggerated expectation that anything they do will become a failure. Accordingly, dysfunctional expectations may drive many psychopathological symptoms and are a prime target for psychotherapies.

## Violating dysfunctional expectations in psychotherapy

In psychopathology, dysfunctional expectations tend to persist. Consequently, this tendency needs to be challenged by experiences that ostensibly violate erroneous expectations. Psychotherapies provide such experiences. Particularly, exposure-based therapies directly target the exact situation/object of the dysfunctional expectation. In exposure therapy, anticipatory anxiety is triggered by the guided exposure of the patient to a threatening stimulus/situation. By means of exposures, current expectations are challenged: a threat signal loses its predictive value and the behavioral response toward the feared stimulus decreases^10,11^. Such effect relies on disconfirmation processes, where the expectation toward a specific stimulus is violated by the surprising absence of the anticipated threat^12^. Because exposure therapy is so explicitly focused on violating expectancies, much of the research on dysfunctional expectations has been done in the context of fear and anxiety.

The positive effects of exposure-based therapies on fear levels can readily be modeled experimentally using extinction learning paradigms. Within these paradigms, first a conditioned stimulus (CS) is repeatedly presented in association with an aversive unconditioned stimulus (US). These contingencies will promote the acquisition of a CS–US association, such that the presentation of the CS alone becomes sufficient to elicit a conditioned (fearful) response (CR). Next, this fear can be extinguished by the repeated presentation of the same CS in the absence of the US. Historically, these procedures have been used to investigate the potential processes underpinning fear reduction. In particular, fear reduction during repeated exposures to the CS (e.g., extinction-based treatments for anxiety) has long been explained by reference to habituation processes. Habituation models consider fear reduction as an essential precursor of long-term therapeutic benefit. In line with this notion, compared to animals that extinguish fear quickly, slow extinguishers are more vulnerable to relapse^13^. However, other work has shown that the amount of fear reduction obtained by the end of extinction training or exposure treatment is not predictive of fear levels at follow-up^14,15^. A clinical improvement in contamination fear after exposure treatment, for instance, is not predicted by the degree of behavioral or physiological fear at the end of such treatment^14^.

More recently, novel safety learning emerged as an additional process underpinning fear extinction^16^, and its long-term retention^17^. This learning process is elicited by novel experiences of the previously conditioned stimulus and the (unexpected) absence of the feared outcome (which constitutes the prediction error, see below). These experiences allow for the formation of a new safety memory regarding the CS, so that the CS now signals the absence of the aversive US (CS → noUS). This new memory representation will henceforth compete with the original threat memory (CS → US) and inhibit fear responding^6,18^. Likewise, exposure treatment is now thought to lay down a novel safety memory that associates a feared situation with the absence of danger. Accordingly, exposure therapy outcomes seem to benefit more from strategies that maximize safety learning processes than from those that promote habituation to threat^19,20^.

## Dysfunctional expectations are resistant to change and prone to relapse

Studies on fear extinction highlight that many anxiety patients or anxious subjects show delayed extinction learning^21,22^. Accordingly, within exposure-based therapies, only about 49.5% of anxious patients show an improvement at the end of treatment^23^. These data suggest that some patients might have difficulties acquiring the information conveyed by the therapeutic experience that the feared stimulus or situation is actually ‘safe’ (e.g., successfully engaging in a social conversation does not lead to novel safety learning that social conversations usually are not embarrassing). Arguably, this lack of extinction learning may reflect a deficit in the prediction-error driven learning process that is critical for the acquisition of safety.

Studies on fear extinction have also indicated that the ability to retrieve fear extinction memories in anxious patients is impaired^24^. Fear extinction memories have to be stored in long-term memory (consolidation) and activated when needed (retrieval); a failure in either of these processes may lead to a return of fear. But, even in healthy individuals, a return of fear can be observed under certain test conditions^25,26^. A return of extinguished fear responding can occur when the time has passed since the end of extinction training^27^; when the extinction context changes to a new or back to the conditioning context^25^; when an unexpected and unsignalled US is presented after extinction (reinstatement)^26^.

Arguably, this characteristic of fear extinction memories has evolutionary benefits, as it makes an individual be cautious rather than rash, in line with a ‘better-safe-than-sorry’ strategy^28^. However, in clinical anxiety such characteristic might be exacerbated. Accordingly, many patients show a relapse in symptomatology after therapy, which has been attributed to difficulties in ‘retrieving’ extinction in addition to ‘acquiring’ extinction^11^.

Clearly, delayed acquisition and impaired retrieval of fear extinction memories point to a necessity to develop new strategies to induce effective and more durable behavioral change. In this review, we address this challenge by considering the similarities between expectancy violation processes and prediction error signaling, in order to merge recent trends in these clinical and fundamental fields of research and to inspire the development of novel adjuncts to psychotherapy.

## Prediction error captures the learning component of expectancy violation

Formal learning theories specify how violations of prior expectations lead to updating of expectation values and induce behavior change. Pavlovian learning theories, such as the Rescorla–Wagner (RW) model^29^, and reinforcement learning theories^1^ highlight how the ‘surprising’ aspect of these violations represents the key ingredient of a learning process. The level of surprise, or the prediction error (PE) signal, has been mathematically formalized as the mismatch between experienced and expected outcome. The RW model describes the result of such a mismatch as a change in the associative strength between two stimuli (CS → US), while reinforcement learning theories extend this notion to ‘action values’ that denote the associative strength between an action and its outcome (action → outcome). Both explain how mismatches induce new learning, lead to change behaviors, and to the updating of expectations. In the present manuscript, we focus on the stimulus → outcome association, applying the basic RW model to fear extinction learning.

The RW model is classically used to explain the strengthening and weakening of stimulus → outcome associations, which is particularly relevant within the context of fear reduction. The RW equation is expressed by the formula: ∆*V* = *α*(*λ* − VΣ)^29^. In this formula, ∆*V* specifies the change in associative strength on a particular learning episode or trial; *λ* refers to the maximum magnitude of the US; *V*_Σ_ is the sum of the associative strengths of all the CSs present on that particular trial, and *α* is a learning-rate parameter (proportional to CS intensity). In the specific case of fear extinction, an expected US is suddenly omitted after the presentation of the CS, which triggers a negative PE that counters the previously learned CS → US association. The mismatch between actual absence and expected delivery of the US is captured in the Rescorla–Wagner model by subtracting the CS → US associative strength from a zero value (representing the absence of the US; *λ* = 0 in the RW equation). This negatively signed PE serves to decreases the strength of the CS → US association in the original RW model; however, later developments in fear extinction learning have yielded a reformulation of the model in which the unexpected absence of the US triggers a novel memory representation of ‘noUS’ and a corresponding positive (reward) PE signal. This positive PE governs the development of a separate, CS → noUS association that competes with the CS → US association for behavioral control^29,30^. This adjustment to the original RW model allows explaining how fear of a CS can return after extinction, because retrieval of the CS → US association is suppressed but the strength of the association as such remains unchanged. Within the context of exposure-based therapy, repeated exposure to a feared situation in the absence of expected harm may generate a positive PE (expectancy violation) that drives new safety learning and counters the erroneous expectation that underlies dysfunctional fear and avoidance behaviors. Thus, the success of safety learning depends on the level of expectancy violation and positively signed PE achieved during the exposure intervention. It is for this reason that expectancy violation strategies aim to keep threat expectancy levels high during each exposure trial, which is radically different from older habituation strategies that strive to reduce fear and threat expectancy during exposure^20^.

## Prediction errors rely on dopamine signaling in the mesolimbic pathway

Error-based learning is strongly modulated by specific neurotransmitters in our nervous system, in particular dopamine^31^. Especially the mesolimbic dopaminergic pathway shapes learning processes by coding PE signals. Extensive research on reward learning highlights dopamine as a neurotransmitter carrying information related to expectations^1^ and to the outcome value of rewards^32^. Numerous studies have indicated that rewarding stimuli, like food delivery, trigger a phasic burst of activity in dopaminergic neurons within the ventral tegmental area (VTA) and the subsequent release of dopamine in the nucleus accumbens (NAcc). Critically, the degree of this dopaminergic activity corresponds to the magnitude of the mismatch between expected and received reward, and to other properties of reward processing (e.g., delay and probability of the reward)^1^^,^^33–35^. Conversely, a decrease in PE (i.e., a reduction in the mismatch between expected and received reward) goes along with a decrease in phasic mesolimbic dopamine response^36^. Here we focus mostly on the role of phasic dopaminergic signaling in encoding the magnitude of the PE, because it is this property that reflects the level of ‘surprise’ associated with specific unexpected events, such as disconfirmations in psychotherapy.

Critically, recent studies have shown that mesolimbic DA is not only involved in coding unexpected rewards but also unexpected omissions of punishment which is directly relevant for fear extinction learning^37,38^. Given that many forms of psychotherapy rely on violating erroneous expectations of negative outcomes, these results suggest that dopamine could also play a role in the learning processes that mediate behavioral change in psychotherapy. Therefore, we review in detail the animal and human studies that have implicated dopamine in fear extinction learning and we investigate its specific role in PE signaling.

### Animal studies

#### Expectancy violations trigger the release of mesolimbic dopamine

Studies in rodents have indicated a time-dependent effect of DA on the acquisition of fear extinction learning. In-vivo microdialysis shows a basal increase in DA and noradrenaline in mPFC during the first phase of extinction training^39^. Accordingly, other studies showed that, similarly to positive rewards^40^, increased release of mesolimbic dopamine has been shown during instrumental avoidance learning^41^ and when a punishment (pain) terminates^42^. A recent study pinned down the critical role of dopamine by demonstrating that dopaminergic neurons in VTA detect the omission of an expected unpleasant US. Optogenetic inhibition of DA neurons at the time of US omission disrupted extinction, demonstrating that especially VTA neurons projecting to the NAcc shell are necessary for the acquisition of fear extinction^38^. This finding is in line with a parallel study that demonstrated how firing of DA neurons during early omission in VTA is both necessary and sufficient for fear extinction learning: whereas optogenetic inhibition of VTA DA neurons at the time of US omission prevents fear extinction acquisition, activation of the same neurons accelerates it^37^. This is in line with the idea that unexpected omissions of aversive events are rewarding and coded as a positive PE by dopaminergic neurons, which then produces new learning about when omission of the US can be expected (i.e., safety learning). Taken together, these results provide important insights into the role of mesolimbic dopamine in the generation of new safety memories^43^. Obviously, this evidence carries important clinical implications for therapies based on expectancy violation procedures, as it suggests that pharmacological manipulations of phasic dopamine levels might enhance the acquisition of fear extinction.

#### Augmenting tonic DA levels improves the acquisition and consolidation of fear extinction

A multitude of animal studies indicates that the consolidation of fear extinction is mediated by DA-levels in the amygdala (AMY) and its intercalated neurons, the medial prefrontal cortex (mPFC), and the hippocampus^44^. In rodents, a systemic increase in extracellular DA-levels through the administration of methylphenidate hydrochloride (MPH), a DA transporter blocker (which, of note, can also increase noradrenaline levels^45^), can promote fear reduction during extinction sessions (indicative of enhanced acquisition of a novel safety memory) when administered before extinction training, and increase extinction retention when administered either before or after the training^45^. Similarly, pre-extinction hippocampal CA1 infusion of MPH in rats that otherwise do not show fear extinction, boosts fear reduction during extinction and enhances its retention through β-adrenergic and D1 receptors^46^. The post-extinction administration of MPH in CA1 does not enhance extinction retention, suggesting that MPH modulates acquisition rather than consolidation of novel safety memories^46^.

Several studies also investigated the effects of other DA enhancers on fear extinction learning, such as l-DOPA. l-DOPA is an indirect dopamine precursor that, different than dopamine itself, can cross the blood–brain barrier; in the brain, l-DOPA is converted into dopamine by the enzyme aromatic l-amino acid decarboxylase^47^. It has been shown that in animals, the post-extinction administration of l-DOPA reduces the return of fear and promotes elevated vmPFC and reduced AMY activity during a delayed test^48^. This is in line with a recent animal study showing that temporarily inhibiting downstream IL-BLA projections during the acquisition of fear extinction impairs extinction memory retrieval^40^. In mice expressing extinction deficits (29S1/SvImJ, S1 which show intact fear learning but impaired acquisition of fear extinction and its consolidation), systemic injection of L-DOPA improves the acquisition of fear extinction (if administered before extinction training) and its consolidation (if administered after extinction training)^49^. Additionally, once fear extinction is acquired, dopamine-related activity in mPFC (and not in NAcc) underlies its long-term retention^50^. Accordingly, rats with a lesion at the level of the vmPFC have been shown to be unable to express fear extinction during a subsequent test (for an overview, see ref. ^10^), while increased levels of bursting of IL neurons were observed to be correlated with extinction recall in extinguished rats^51^.

Of note, studies in stressed rodents (which are often used to simulate anxiety disorders) have shown that fear extinction retrieval deficits following stress are associated with the presence of low extracellular dopamine levels in fear-related circuits^52^. Although such results have to be taken with caution (see Box 1), it seems that increasing tonic dopamine levels, especially in prefrontal regions of the brain, could potentially facilitate the acquisition and consolidation of extinction, also in the presence of a fear extinction deficit. Crucially, however, it is still unknown if DA-based interventions might produce different effects when the baseline DA is higher. Consequently, the individual profile in basal tonic DA must be considered if pharmacological dopaminergic interventions can be efficacy used to increase fear extinction in the context of anxiety. Finally, it remains presently unclear whether a pre-retrieval pharmacological manipulation of dopamine levels might modulate the capacity to retrieve fear extinction.

#### Blocking DA receptors interferes with the acquisition and consolidation of fear extinction

D1 and D2 G-protein-coupled receptors (D1R, D2R) govern a large number of DA-dependent learning processes via long-term potentiation and depotentiation and therefore, via neuroplasticity. Their density varies along the dopamine system: mRNA encoding D2R (conveying genetic information from DNA to the ribosome) is highly present in VTA^53^, and D2 and especially D1 receptor genes are highly expressed in PFC^54^ and hippocampus^55^. Several studies investigated the contribution of these DA receptors in fear extinction.

Animal findings show that pre-extinction injection of D2R antagonists (sulpiride) in the basolateral amygdala (BLA) delay fear extinction during training and its long-term retention, while pre-extinction infusion of D2R agonists (quinpirole) in the same brain area increases the acquisition of fear extinction and its long-term retention^56^. Additionally, pre-extinction injection of a D2R antagonist (raclopride) in IL impairs later retrieval of fear extinction in rodents without affecting its acquisition^57^. A similar study in rodents, however, highlights how effects of pharmacological manipulations of DA receptors in IL are age-dependent, with quinpirole effects on long-term fear extinction present only during youth^58^. Similarly, pre-extinction blocking of D2R decreases the positive effect of glucocorticoids on fear memory extinction^59^ although in another study it facilitates extinction 24 h after conditioning^60^.

More evidence for an involvement of D2R comes from a study that shows that extinction training in mice decreases D2R mRNA in the IL^61^. Hence, it seems that (still unspecified) changes in D2R-related DA activity interact with the acquisition of fear extinction and possibly with its consolidation and retrieval; yet, such changes might exert opposite effects depending on whether pharmacological manipulations are administrated locally (e.g., IL) or systemically. Furthermore, especially from a translational view, it is important to bear in mind the possibility that agonistic and antagonistic effects on D2R (or other DA receptors) might have different consequences on the consolidation and retrieval of fear extinction when administered pre- versus post-extinction learning.

**Fig. 1:**
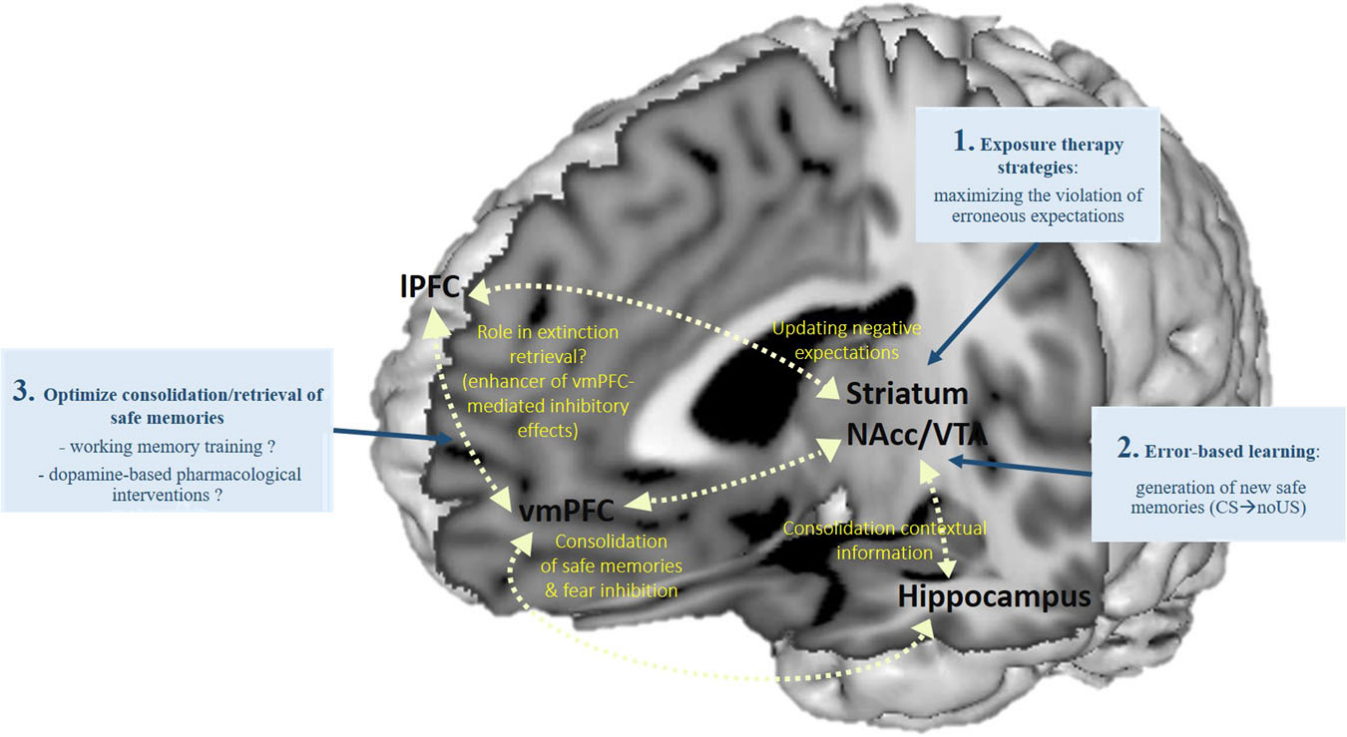
Dopamine modulates the encoding and consolidation of fear extinction: implications for expectancy violation-based therapies. Expectancy violation-based therapies, such as exposure treatment, disconfirm negative expectations through exposure to fear-eliciting situations in the absence of the feared outcome (step 1). This procedure generates a DA-based PE at the level of the NAcc and VTA (mesolimbic brain areas). This signal drives the acquisition of new safety memories (step 2). The phasic DA signal might involve mostly D2R, which interacts with tonic dopamine processes in other brain areas. Dopaminergic transmissions (yellow dashed lines) from midbrain regions to different regions of the prefrontal cortex might be responsible for the updating of negative expectations (or goal-relevant representations) of threat (vmPFC) and for the retrieval of fear extinction memories (possibly with the involvement of DA in lPFC). This process might involve principally tonic D1R signaling in the prefrontal cortex and in the hippocampus, two key areas for the consolidation of fear extinction memories. Future studies should further investigate whether the dopamine-based intervention (especially l-DOPA administration) as well as WM training can promote fear extinction retrieval and thus long-term gains of successful exposure treatment psychotherapy (step 3).

The evidence from animal studies seems to indicate that reduced D1R activity is associated with worse acquisition and consolidation of extinction, although results have to be interpreted cautiously. In mice, a genetic reduction in D1R is linked to a delayed acquisition of fear extinction^62^ and a pre-training blockade (antagonist SCH23390) of D1R in BLA reduces the acquisition of fear extinction but not its consolidation^63^. On the other hand, fear extinction consolidation decreases when the pre- or post-administration of the same antagonist is applied in IL^63^. A previous study also found that activating D1 receptors in the dorsal striatum and the substantia nigra during fear extinction enhances exclusively its consolidation^64^. Given that D1R in the hippocampus is also involved in the formation of long-term fear memory (LTM)^65^ and in contextual fear conditioning^66,67^, recent studies have investigated the role of D1R in the context of fear extinction and its consolidation. Results from these studies show that facilitation of fear extinction (by novelty) is mediated by D1R dopamine-dependent hippocampal activity^68^, and that pre-extinction blockade of these receptors reverses the positive effects that pre-extinction infusion of MPH exerts on contextual fear extinction learning and (possibly) its retention^46^. The role of hippocampal D1R in consolidating fear extinction memories is further supported by the fact that long-term memory (LTM) of fearful experiences depends on activation of VTA/CA1 hippocampus dopaminergic connections, mainly involving D1R and mediated by brain-derived neurotrophic factor^69^ (Fig. 1). Although these results suggest overall a fear extinction improvement by enhancing D1R-related activity, it should be noted that contrasting findings have also been reported in the literature for both acquisition^63,70^ and consolidation of fear extinction^58^. For example, it has been shown that, in a small sample of male mice, blocking D1R in IL right before a reinstatement of the fear test in a new context B actually prevents the return of fear^71^. It is important to note, however, that reinstatement testing is quite complex and involves a context conditioning mechanism: unsignalled presentations of the aversive shock lead to the formation of a context → US association, which then retrieves the CS → US association and leads to the return of fear. It is possible, therefore, that D1R-blockage weakens the return of fear by blocking the context conditioning mechanism rather than enhancing extinction retrieval per se. To further investigate the role of prefrontal D1R in the reinstatement of fear, future studies should test the presence of this effect also when D1-blockage is applied after reinstatement procedures, before the test.

D3 receptors have not yet been studied in the context of fear extinction, but rodent studies found that antagonizing D3 receptors in BLA decreases anxiety-like symptoms^72^, also in an animal model of PTSD^73^. Similarly, D3R-deficient mice show reduced freezing during contextual fear conditioning and decreased anxiety^74^. In healthy humans, on the other hand, augmented prefrontal D3R availability is linked to a higher amygdala response to aversive cues^75^. Clearly, there is a link between D3R and fear expression, but their involvement in fear extinction learning remains to be investigated.

In summary, the specific roles of D1R, D2R, and D3R in fear extinction remain to be further clarified (Box 1). However, in rodents, mesolimbic and prefrontal DA levels modulate the acquisition and consolidation of fear extinction learning; on the one hand, this evidence supports the great utility in studying the effects of dopamine-based pharmacological interventions to boost fear extinction, such as l-DOPA, Neuropeptide S (probably due to its enhancing effects on mPFC dopamine), methylphenidate, or sulpiride (for an overview see ref. ^76^). These dopaminergic manipulations could be then potentially used during or after therapy to enhance the long-term effects of exposures exercises. On the other hand, it remains still unknown if dopaminergic manipulations via systemic administrations can interfere with DA transmission in other brain regions, causing alterations (side effects) in other DA-dependent cognitive functions.

Box 1 Fear extinction and dopamine: current challenges from animal research. The effects of DA-receptor manipulation on the acquisition and consolidation of fear extinction might vary in relation to:

- the brain area(s) under investigation (DA receptors might play a different role depending on their location in the brain)^53^;
- the age of the sample under analysis (pharmacological antagonists and agonists might affect DA receptors in a different manner along the lifespan);
- the still unknown actions exerted by D1R, D2R, and D3R in different parts of the brain;
- the individual basic level of DA;
- the different density and localization of D1R, D2R, and D3R receptors across the brain;
- time-dependent effects of the pharmacological manipulation (e.g., pre- or post-training);
- the confounding effects on other neurobiological systems (e.g., Noradrenergic system).

This last point represents an important issue given that DA interacts with a variety of other neurotransmitters. As a clear example, the progress of fear extinction is typically evaluated through behavioral changes in animals, often involving movement (e.g., freezing or rate of exploration). However, induced changes in dopamine levels strongly affect the locomotor system^119^ and the motivation to move^120^, in particular when injections are systemic and therefore also reach dopamine-related motor circuits (e.g., substantia nigra and basal ganglia). Although recent studies have attempted to those confounds into account (tests are usually performed after 24 h since the DA-manipulation), this still represents a potential issue in interpreting and translating animal findings and in interpreting human findings.

### Human studies: mesolimbic and prefrontal dopamine is involved in expectancy violation and fear extinction consolidation

Few studies have investigated how DA signaling mediates fear extinction learning in humans. In line with the animal findings, a functional polymorphism in the DA transporter (DAT) gene, (which regulates extracellular DA levels in the striatum, and presumably controls extracellular DA during phasic DA release), affects the acquisition of fear extinction, with DAT1 9R carriers showing a higher extinction learning rate (corresponding to learning rate parameter *α* in the RW equation) and higher hemodynamic responses to US omissions in the ventral striatum than non-9R carriers^77^. This result adds credit to the application of the dopaminergic theory of PE^1^ to the acquisition of fear extinction in humans. Other findings indicate that subjects carrying two met alleles of the gene codifying the enzyme catechol-O-methyltransferase (COMTval158met polymorphism) and therefore displaying a higher extracellular DA profile especially in pre-frontal regions, also fail to extinguish fear^78^. Theoretical models of the COMT met allele describe this condition as involving a reduction in phasic DA in sub-cortical regions (potentially causing a restricted flexibility of activation states, such as those involved in PE coding), coupled with a higher tonic extracellular prefrontal DA level and an increased D1 cortical activity (potentially causing a hyper-stability of cortical activation states, yielding rigid behavior)^79^. Consequently, on the basis of the available findings on fear extinction and theoretical DA models regarding COMT and DAT polymorphisms, we suggest that reduced striatal DA activity might impair the acquisition of fear extinction in humans. This suggestion, however, remains speculative and rests merely on the complex DA profile associated with these polymorphisms.

Complementary to reduced striatal DA transmission, a met allele advantage for tasks requiring cognitive stability (e.g., online maintenance of relevant information) has sometimes been reported^80^ (although findings are inconsistent, see ref. ^81^). Specifically, optimal D1R stimulation in PFC networks is thought to facilitate cognitive stability by maintaining information ‘online’ and protecting this information against interfering experiences^3,82^. Conversely, val carriers have been suggested to display better performance in tasks requiring cognitive flexibility (e.g., task switching^83,84^), increased D2-mediated phasic DA transmission, and decreased D1-mediated cortical DA transmission^79^. Given that fear extinction learning first requires the processing of expectancy violation (and thus, cognitive flexibility) for its acquisition and subsequently requires cognitive stability for its consolidation and retrieval, the COMTval158met polymorphism might hold promise for future research on DA-based mechanisms of fear extinction learning. As an example, it would be interesting to investigate whether COMT val or val/val carriers may have an intact capacity to acquire extinction but a reduced capacity to consolidate and retrieve fear extinction.

The evidence for an involvement of prefrontal dopamine in the consolidation of fear extinction is more straightforward. Like in animals, the post-extinction administration of l-DOPA decreases the later return of fear^48,85^. Furthermore, the degree of spontaneous replay of activation patterns observed during US-omission in vmPFC predicts extinction memory retrieval, an effect that is enhanced by the post-extinction administration of l-DOPA^85,86^. Of note, compared to a control group, elevated vmPFC neural activity (but not fear reduction in skin conductance) during a return-of-fear test was found 1 week after post-extinction l-DOPA administration^87^, indicating that l-DOPA has long-term effects on the activity of brain areas involved in fear extinction retention. Importantly, post-extinction l-DOPA administration successfully reduces fear levels during a later retrieval test only if extinction is effective (i.e., produced a complete reduction of conditioned fear by the end of the extinction training)^85^.

To summarize, further studies should investigate whether the modulation of phasic DA levels can influence the acquisition of fear extinction also in humans. Meanwhile, dopamine-based interventions do clearly emerge as potential adjuncts for long-term gains after successful psychotherapy. To sharpen the scientific knowledge that could support the application of DA-enhancers in exposure treatment, future pharmacological studies on fear extinction should additionally investigate whether the acquisition of fear extinction and/or its consolidation is impaired in the case of aberrant basal dopaminergic activity in PFC, as might be present in psychiatric disorders (Box 2).

## Dopamine in psychotherapy: boosting the effects of expectancy violation

The studies described above carry implications for psychotherapies that use expectancy violation techniques to change maladaptive behaviors. Most of the experiments described indeed mimic the dynamic of a classical exposure exercise. Given the central role of mesolimbic dopamine signaling in processing PEs and updating expectations, and given that prefrontal dopamine seems to be linked to the successful consolidation of fear extinction memories, we propose that expectancy violation techniques in psychotherapy might benefit from including DA-based interventions in three different moments: during the acquisition of new safe memories (at the moment of PE), during the subsequent consolidation, and at the time of intended retrieval of those memories^48,85^.

With regard to the acquisition, such interventions could take the form of the administrations of drugs that modulate phasic dopamine at the moment of scheduled expectancy violation. In exposure treatment, this can be accomplished by guiding a patient through a feared situation in the absence of the expected aversive event. From the basic research described above, we expect that targeted pharmacological interventions may lead to stronger acquisition of the new safety experiences that can then more strongly counter the existing fear associations. Such interventions fit with an inhibitory model of fear extinction, according to which fear reduction from exposure treatment is mainly obtained through novel safety learning. However, it remains unknown which agonist and/or antagonist would exclusively target phasic dopamine in the VTA/ventral striatum during exposure. To date, pharmacological manipulations of dopamine levels in humans influence both phasic and tonic dopamine signaling, making it impossible to separately optimize striatal and prefrontal dopaminergic fear extinction processes involved in acquisition and consolidation, respectively. On the basis of the current knowledge base on dopaminergic signaling, therefore, we here emphasize behavioral options for keeping US-expectancy levels high during exposure, in order to maximize surprise (unexpected US omissions) and enhance the phasic release of DA (Fig. 1). Inducing a high PE during each exposure exercise might indeed lead to a stronger inhibition of clinical anxiety via safety learning^17^. Additionally, within the animal research, emerging results seem to indicate that diet manipulations (e.g., acute fasting, diet restrictions) might also serve to increase phasic dopaminergic outcome in reward-learning areas, such as VTA and NAcc^88–91^. For example, food restriction has been shown to increase mRNA levels of tyrosine hydroxylase (an enzyme involved in the synthesis of DA) and DA transporter in VTA of male rats^90^, suggesting that food restriction might sensitize the mesolimbic system.

With regard to the consolidation and later retrieval of safe memories, dopamine-based pharmacological interventions, such as l-DOPA administration after therapy, effectively reduce the return of fear in healthy individuals and are promising for clinical use. However, the effects of l-DOPA might occur only if substantial fear extinction has been achieved during the session^85^. Also, clinical trials testing the effects of l-DOPA in the presence of pre-existing alterations in PFC dopamine activity are urgently needed. This is an important step to bridge the current evidence in healthy humans and future clinical application of DA-enhancers in patients, given that in clinical conditions DA levels may not correspond to a functional DA profile (Box 2). In those circumstances, l-DOPA administration might even affect treatment outcomes negatively. Furthermore, studies should investigate the effect of l-DOPA specifically on the ability to retrieve fear extinction memories and to counteract the retrieval of prior threat expectations.

Box 2 Dopamine and fear extinction learning in anxiety: a problem in the acquisition or in the consolidation of fear extinction? Positron emission tomography (PET) and single photon emission computerized tomography (SPECT) studies investigated the role of DA in clinical anxiety, often in patients with symptoms of social anxiety^121–125^. A recent review of these molecular neuroimaging studies points to the presence of an alteration in striatal DA functioning in anxious patients, although the findings are not always consistent across studies^126^. In light of the role that striatal DA has in the acquisition of fear extinction in animals, we suggest that altered striatal DA functioning in anxious patients may be associated with a potential decrease in their ability to learn from errors (e.g., from unexpected US-omission). Future studies in clinical (anxious) populations should therefore examine whether the presence of striatal DA alterations is associated with difficulties in the acquisition of fear extinction (e.g., impaired safety learning). In this respect, results from a recent fMRI study involving individuals with a diagnosis of specific phobia showed how high vmPFC activation during US-omissions (together with a trend found in the NAcc during the same conditions) was predictive of a reduction in clinical symptoms after exposure therapy^127^. These results indicate that, in the presence of clinical anxiety, higher prediction-error-related signaling (crucial for learning) is associated with better therapeutic outcome. Additionally, it has been recently shown that a reduction in anxiety-related symptoms after cognitive behavioral therapy (CBT) was negatively associated with increased D2R receptor binding in the mPFC and hippocampus of individuals with social anxiety^128^, and elevated D2R receptor availability was found in the OFC and dlPFC of patients with the same diagnosis^129^. These results indicate the presence of a prefrontal DA alteration in anxious individuals. On the basis of the evidence that DA transmission in prefrontal regions of the brain is crucial for the consolidation of fear extinction, we suggest that future studies on clinical anxiety should also investigate whether the presence of aberrant prefrontal DA activity is associated with the impaired ability to consolidate and retrieve fear extinction memories and alterations in WM capacity.

## Optimizing prefrontal dopamine modulation: a potential role for working memory?

Although prefrontal dopaminergic manipulations after successful fear extinction procedures are emerging as a promising adjunction to maintain the long-term outcomes of exposure, these systemic manipulations do not guarantee specificity in their effects. This is particularly true in light of the fact that dopamine acts as a neuromodulator^92^ for other important brain functions: reinforcement learning, motivation, executive functions, motor control, arousal, and reward, just to name a few. Consequently, developing behavioral strategies to optimize prefrontal dopamine modulation during fear extinction could provide safer and more specific advantages as adjuncts to psychotherapy^93^.

Positron emission tomography studies indicate that working memory (WM), the capacity to retrieve and keep goal-relevant information online and to use it to guide adaptive behavior^94–96^, relates to dopaminergic activity in PFC^97^. Some evidence that WM training increases activity in prefrontal regions of the brain^98^ and cortical DA already exists (see below)^99^. Especially, the lPFC is a brain region rich in DA projections and described by influential neural models of cognition as heavily involved in attention and working memory capacity^96,100,101^. Additionally, human theories of fear emotion regulation suggest that the lateral PFC could enhance the inhibitory effects that the vmPFC exerts on fear levels during extinction (e.g., by suppressing amygdala reactivity)^102^. Since the presence of an elevated functional connectivity between lPFC and vmPFC, lPFC has been recently used as a target in transcranial magnetic stimulation (TMS) during extinction learning. The results from this TMS-study showed an enhancement of fear extinction recall 1 day after the intervention^103^. Based on these emerging lines of evidence, we propose that behavioral strategies that enhance WM capacity could serve to optimize dopamine-related activity in lateral PFC and, consequently, improve long-term fear extinction retrieval.

To date, no study has investigated a direct dopaminergic link between WM capacity and the ability to retrieve safe memories. Nevertheless, such dopaminergic link is suggested by indirect evidence. First, WM capacity is positively associated to a higher fear inhibition^104^; second, subjects high in anxiety show poor safety learning and concomitant low memory capacity^105^; third, a tendency in anxious individuals to misallocate WM resources to threatening distractors has been linked to enhanced reactivity of amygdala nuclei^106^; finally, pathological anxiety has been linked to meso-corticolimbic DA alterations^107^. In the next paragraphs, on the base of recent influential dopamine-based models of cognition, we develop a theoretical framework that can set the stage for future studies to elucidate the potential role of WM in maintaining long-term gains of exposure therapies.

### Working memory capacity and the meso-cortico-limbic DA system

A hypothetical link between working memory, meso-corticolimbic DA, and individual ability in retrieving fear extinction might be re-framed within recent theoretical models of dopamine-action on other cognitive domains. For these models, the midbrain–PFC system seems to be involved in maintaining an equilibrium between ‘updating’ representations in working memory (via PE-related midbrain phasic dopamine and D2R) and keeping such PFC-representations ‘stable’ in the WM buffer (mediated by prefrontal D1) despite distractions^108,109^. Indeed, D1-receptor antagonists cause impaired performance during delayed response tasks that measure the ability to keep goal-relevant information online^109^. Moreover, as within the context of impaired fear extinction retrieval, this impairment can be reversed by l-DOPA administration^3,70^. Additionally, positron emission tomography studies show that striatal^110^ and prefrontal dopaminergic functions^111^ are related to and predicted by the individual WM profile. Consequently, WM capacity seems to reflects this complex (midbrain–PFC) oppositional dopaminergic dual system.

Given this link, working memory capacity is also emerging as a potentially useful proxy for DA functioning—measuring prefrontal DA levels directly is very challenging^3^. Additionally, prefrontal and midbrain dopamine interacts with tonic hippocampal DA (mainly mediated by D1R) for the generation of long-term episodic memory. Consequently, it is not surprising that WM also promotes the formation of long-term episodic memories^112,113^ via lateral PFC and hippocampus activations^113^. However, although dopamine seems to ‘tune’ learning across different brain areas (from updating till long-term storage of information), the specific steps of such learning mechanism remain uncertain, especially within the context of fear extinction. Crucially, future studies aiming to improve exposure-based therapies, should investigate whether potential positive effects of WM training on prefrontal dopamine and fear extinction consolidation exist.

### Future directions: working memory training within the context of exposure therapy

It remains unknown whether non-pharmacological interventions aimed at strengthening WM might also help to improve the ability to retrieve safe memories when needed. This ability is crucial, since it might reduce negative expectations and rigid behaviors like excessive avoidance, and hence favor long-term therapeutic gains. Within an exposure therapy context, it is noteworthy that some evidence suggests that WM capacity is trainable, with such training (35 min daily for 5 weeks) yielding changes in cortical DR1, as measured via PET before and after training^99^. Interestingly, training of WM and other basic cognitive processes of executive functions has already been adopted successfully to increase response inhibition in obesity, resulting in increased retention of weight loss after a cognitive behavioral therapy-based weight loss program^114,115^. Given that extinction retrieval, like any other form of episodic memory retrieval, is mediated by working memory activity, WM training could be used to enhance the ability to recall and maintain ‘online’ the extinction memory for the time that is needed to positively influence decision-making.

Critically, performance gains in tasks involving short-term or WM components following WM training seem mostly restricted to ‘near-transfer effects’^116^. Therefore, WM training procedures may be most successful if they specifically target processes that are relevant for and experiential contents that the patient (successfully) acquired during the exposure sessions. Relevant procedures for this may include tasks that require frequent memory updating, affective procedures to enhance retrieval ability, rehearsal exercises, and others^116,117^. Finally, we argue that WM training might improve fear extinction retrieval via prefrontal dopamine modulation. WM training may generate safer effects compared to post-extinction pharmacological DA manipulations (such as l-DOPA^85^). Additionally, by interfering with prefrontal DA-activity in mPFC and lPFC, WM training may generate more specific effects than other non-pharmacological interventions, such as physical activity (e.g., aerobic exercises); the latter seems indeed to primarily increase DA-related activity in the dorsal striatum and basal ganglia^118^, although it remains unclear whether more skilled and motor learning-based activities (e.g., Yoga) might actually induce changes also in prefrontal DA transmission.

## Conclusions

Many forms of psychotherapy involve expectancy violation to induce new learning and behavioral change. Formal learning theory conceptualizes expectancy violation as prediction errors, and empirical studies have linked prediction error-based learning convincingly to mesolimbic dopaminergic signaling. Strategies that maximize dopamine-mediated prediction error signaling might therefore enhance the encoding of new learning experiences in psychotherapy, to change maladaptive behaviors. We propose that to facilitate a patient’s retrieval of beneficial memories laid down in psychotherapy, the effects of dopamine-related interventions (including working memory training) after a successful therapy should be investigated in future clinical trials.
